# What is the potential impact of insecticide treated nets in a semi-arid region of northern Kenya? An investigation of vector populations prior to ITN roll-out in Turkana, Kenya

**DOI:** 10.21203/rs.3.rs-7478426/v1

**Published:** 2025-09-19

**Authors:** Lucy Abel, Samuel Kahindi, David Ekai, Erastus Kirwa, Rebecca Lokwang, Mark Amunga, Evans Omollo, Emmah Kimachas, Millicent Cherono, Linda Maraga, Diana Menya, Andrew Obala, Wendy Prudhomme O’Meara

**Affiliations:** Academic Model providing Acess to Health Care(AMPATH); School of Pure and Applied Sciences, Pwani University; Department of Health Services and Sanitation, Turkana County; Academic Model providing Acess to Health Care(AMPATH); Duke Global Inc; Academic Model providing Acess to Health Care(AMPATH); Duke Global Inc; Academic Model providing Acess to Health Care(AMPATH); Academic Model providing Acess to Health Care(AMPATH); Academic Model providing Acess to Health Care(AMPATH); Moi University College of Health Sciences, School of Public Health; Moi University College of Health Science, School of Medicine; Duke Global Health Institute, Duke University

## Abstract

The malaria ecology of northern Kenya differs from the rest of the country. Transmission is highly seasonal and intense, despite the arid environment and low population density. The region faces several threats to malaria control including identification of significant *P. vivax* infection and an emerging invasive species, *An. stephensi*. Turkana County implemented its first mass bednet distribution in late 2024. Prior to this, we established a surveillance program to understand mosquito density, vector diversity, host preference and contribution to malaria transmission. We captured mosquitoes in rural and peri-urban areas, indoors and outdoots, over one year. Twenty-percent of female anophelines were captured outdoors but *P. falciparum* infection was higher in outdoor collections. *An. coluzzi*, a vector commonly found in West Africa, was the second most abundant species and had the highest *P. falciparum* infection rates. These data will be useful in predicting the impact of ITN distribution in this unique context.

## Background

Turkana County is located at the far northwestern corner of Kenya where it borders Uganda to the west and South Sudan to the north. It was previously assumed to be a low-risk area for malaria transmission due to extremely low rainfall and prone to periodic outbreaks during periods of unusual rain. More recent studies have documented widespread endemic transmission in the region, and have uncovered new challenges to malaria control including the presence of *Plasmodium vivax* and the invasive vector, *Anopheles stephensi* (Markwalter et al., 2022; Meredith et al., 2024; Ochomo et al., 2023)

In response to the growing understanding of the burden of *Plasmodium falciparum* in Turkana, the National Malaria Control Programme implemented the first insecticide treated bednet distribution campaign in Turkana County in October 2024. Prior to this, ownership of ITNs was very low; less than 20% of households in peri-urban areas owned an ITN and in some rural areas, no nets could be found at all(Meredith et al., 2021). Following the campaign, ITN ownership rose to 98% (Menya, personal communications, November 2024).

There are very few areas in sub-Saharan Africa with such high levels of transmission but almost completely naïve to vector control. This, coupled with the identification of *Anopheles stephensi*, prompted a year-long surveillance program to try to understand the potential for ITNs to reduce malaria transmission based on documenting the species of vectors, the seasonality of their abundance, their feeding habits and their infectiousness. Here we describe the results of 12 months of adult mosquito surveillance in both peri-urban and rural areas of Turkana Central subcounty prior to the roll-out of ITNs.

## Methods

### Study area

The study was carried out in Turkana Central Sub-County, in Turkana County located in the northwestern corner of Kenya. Two health facilities, one in the peri-urban area around the county capital of Lodwar and one in a remote area 60 kilometers east of Lodwar were selected for entomological surveillance. Both facilities report seasonally high numbers of malaria cases. Each week, two mRDT positive patients from each facility (4 households total) were randomly selected for entomological surveillance over four consecutive nights in their households.

### Entomological collections

Adult mosquitoes were collected using Biogents Sentinel traps (Batista et al., 2018) attached to 2000amp power banks to enable them to run for 24 hours. Two traps were set per household, one indoors close to the case patient’s sleeping space and one outdoors, near an animal enclosure if present. Sugar and yeast were mixed with water to generate CO_2_ to act as an attractant. The exhaust from the yeast reaction was piped into the trap. Each morning the mosquitoes were collected from the trapping bag and a new bag placed for the following collection night. The power banks were replaced with freshly charged ones and the CO_2_ mixture was replenished each morning when the traps were emptied.

The contents of the trapping bag were sorted and only the mosquitoes were retained. These were packaged in a petri dish and transported to the laboratory in Webuye, Kenya for further processing. In the lab, *Anopheles* and *Culex* mosquitoes were counted by sex and all the female *Anopheles* mosquitoes were morphologically identified to species under a microscope using morphological keys (Coetzee, 2020). A photograph of the major morphological features (head, wings and palps) was taken for quality assurance and confirmation by the senior entomologist. Each female *Anopheles* mosquito was put in a labelled 1.5ml tube. The samples were then issued to the laboratory for molecular analysis.

### Molecular analysis

#### DNA Extraction

*Anopheles* mosquitoes were extracted utilizing the Hotshot DNA Extraction method as described by Truett with modification (Truett et al., 2000). Briefly, mosquitoes were individually placed into wells of a 96-well plate after removing the heads (leaving thoraces intact). Fifty microliters of alkaline lysis buffer were added to each well containing the mosquito sample, and the plate was incubated at 95°C for 30 minutes. An equal volume of neutralizing solution (Tris HCl pH 5.0) was added, and plates were subsequently stored at −20°C.

#### Molecular species determination

Mosquitoes were first identified morphologically. Samples which were identified morphologically as *An. Gambiae s.l.* were tested using molecular methods to distinguish *An. gambiae s.s., An. coluzzi*, and *An. arabiensis*. Multiplex PCR was run on 5 μl of extract following the method of Wilkins et al (Wilkins et al., 2006). Reactions were run on a 1% agarose gel at 100V for 40mins. DNA fragments were visualized under UV light using SYBR safe stain added to the agarose gel. Samples that did not amplify in this assay were tested for *An. stephensi* (Balkew et al., 2021). *Anopheles gambiae s.l.* specimens which did not amplify with primers for these species retained their designation of *An. gambiae s.l.*. Nineteen samples that were identified morphologically as *An. gambaie s.l.* but which did not amplify in our molecular assay were sent to CDC Atlanta for confirmatory sequencing, along with two known samples detected as *An. arabiensis* and *An. coluzzii* for comparison. Samples were sequenced at the COI and ITS2 regions after DNA extraction from a mosquito leg or wing.

#### Plasmodium falciparum detection

1μl of the mosquito gDNA was used for *Plasmodium falciparum* detection. A real-time PCR assay targeting the multi-copy motif in the *P. falciparum* genome called pfr364 was performed (Taylor et al., 2019).

#### Blood Meal Analysis

All mosquitoes, regardless of their observed abdominal status, were tested for the presence of human or animal bloodmeal (Hoffman et al., 2021). The PCR amplifies the 12S ribosomal RNA gene with a product size of 205bp for any vertebrate DNA. Another product of 154bp for human blood meal is also amplified if human genomic DNA is present. The PCR product was then run on a 2% agarose gel.

## Results

Mosquito collection was conducted between January 2024 to Jan 2025 in 170 households for a total of 586 nights. A total of 5217 female mosquitoes were collected of which 49% (n=2,515) were anopheles (Table S1). Most female anopheles were caught indoors (n=2,012, 80.4%) and 94.3% (n=2,371) were trapped in the rural site. 2,502 were tested for *P.falciparum* infection and human or animal bloodmeal.

**Figure 1:**
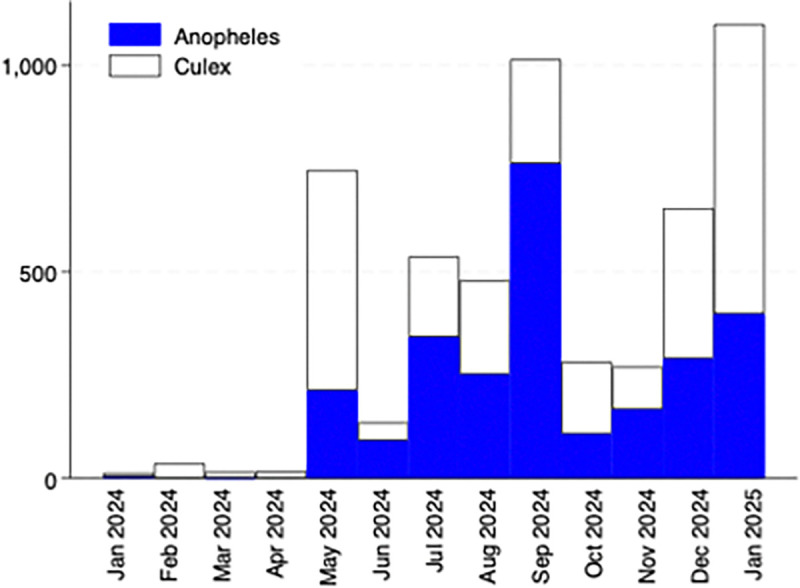
Total female mosquitoes trapped indoors and outdoors by month, shaded by anopheles and non-anopheles.

Female anopheles and non-anopheline mosquitoes were trapped in roughly equal number over the course of the year (Figure 1). The main peak of anopheles mosquitoes was observed in September with a smaller peak in January 2025 just before collection ended. Seasonality was similar for both anopheles and non-anopheline mosquitoes, although non-anopheline also demonstrated an earlier peak in May 2024 and higher density in January 2025.

**Figure 2:**
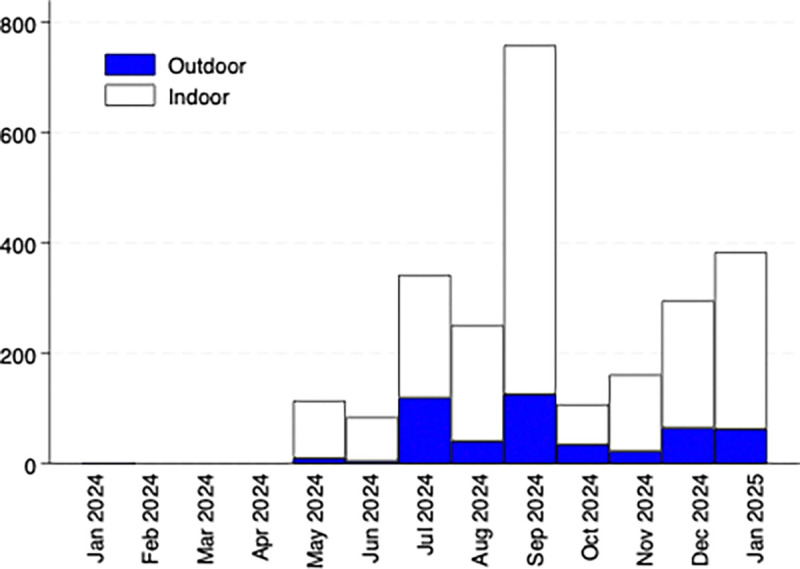
Number of female anopheles mosquitoes trapped indoors and outdoors by month

The majority of female *Anopheles* mosquitoes were collected indoors near indoor sleeping spaces (Figure 2). Indoor sleeping spaces, particularly in the rural area where >90% of mosquitoes were trapped, are most often on the ground within a structure constructed from branches and palm (Table S2). These traditional structures are very porous, have no “eaves” nor doors and windows. Overall, fewer mosquitoes were collected outdoors and these collections had less pronounced seasonality; small numbers of female *Anopheles* were collected outdoors throughout the period from May to January.

Overall, *P. falciparum* genomic DNA was detected in 4.5% of female *Anopheles* (Table 1). The proportion was significantly higher in outdoor collected mosquitoes (p=0.038, chi square). In contrast, the proportion of mosquitoes with human DNA detected was small and not significantly different between indoor and outdoor collected mosquitoes (p=0.089, chi-square). Animal bloodmeal sources were roughly ten times more common. As expected for a passive trap, most mosquitoes were unfed and probably caught while foraging.

Nine different anopheles species were identified. *An. arabiensis* was the most abundant (53.7%), followed by *An. coluzzii* (23.6%). Slightly over 6% of mosquitoes were identified as *An. pharoensis*. Only three adult female mosquitoes were identified as *An. stephensi*. A total of 292 mosquitoes identified morphologically as *An. gambiae s.l.* could not be confirmed as *An. arabiensis, An. gambiae s.s.*, *An. stephensi*, or *An. coluzzi*. A small subset of these (19) were sent for sequencing (10 returned sequences at COI and 6 returned sequences at ITS2) and were identified as either *An. arabiensis* (n=4)*, An. pharoensis* (n=1) or *An. coluzzii (*n=3). Three were only identified at the complex-level *(An. gambiae s.l.)*. However, 40% (8/19) did not return usable sequence data likely due to degraded samples, possibly as a result of being collected and stored at high ambient temperature.

Four of the vector species we identified were infected with *P. falciparum*. Twice as many *An coluzzii* were infected with *P. falciparum* compared to *An. arabiensis*. *An. pharoensis* and *An. funestus* were also infected. All the species represented by at least 10 individual mosquitoes were confirmed to feed on non-human mammalian hosts. Among fed mosquitoes, 4–10 times more meals were identified as non-human bloodmeals than human. *An. arabiensis, An. coluzzii* and *An. pharoensis* were confirmed to feed on humans. No *An. funestus* had a human bloodmeal, although 2/18 were *P.falciparum*-infected thus implicating them in local transmission.

**Table:** 

	Bloodmeal					*P. falciparum* infection			
	Human	Non-human	None	Total^1^	p	Infected	Uninfected	Total	P
N	53 (2.2%)	550 (22.6%)	1,829 (75.2%)	2,432	<0.001	113 (4.5%)	2,389 (95.5%)	2,502	<0.001
*An. arabiensis*	35 (2.7%)	405 (30.9%)	870 (66.4%)	1,310		44 (3.3%)	1,304 (96.7%)	1,348	
*An. coluzzi*	13 (2.3%)	70 (12.1%)	494 (85.6%)	577		47 (7.9%)	549 (92.1%)	596	
*An. gambiae s.l.*^2^	4 (1.4%)	43 (14.3%)	244 (84.3%)	291		15 (5.1%)	272 (94.9%)	292	
*An. pharoensis*	1 (0.7%)	18 (12%)	131 (87.3%)	150		2 (1.3%)	158 (98.7%)	160	
*An. funestus*	0 (0.0%)	6 (33.3%)	12 (66.7%)	18		2 (10.5%)	17 (89.3%)	19	
Undetermined	0 (0.0%)	7 (10%)	63 (90%)	70		3 (4.2%)	68 (95.8%)	71	
Other ^3^	0 (0.0%)	2 (15.4%)	14 (84.6%)	16		0 (0.0%)	16 (100%)	16	

1. 70 samples gave ambiguous results in the bloodmeal analysis and are not included in the table

2. Mosquitoes identified morphologically as An. gambiae s.l. but which did not amplify with An. arabiensis, An.coluzzi, or An. gambaie s.s. primers are listed as *An. gambiae s.l*.

3. Other species identified include *An. stephensi* (n=3), *An. rufipes* (n=2), *An. coustani* (n=4), and *An. demeilloni* (n=7). None had *P.f*. or human bloodmeal detected. Two *An. coustani* and one *An. stephensi* were positive for non-human bloodmeal

1. 70 samples gave ambiguous results in the bloodmeal analysis and are not included in the table
2. Mosquitoes identified morphologically as An. gambiae s.l. but which did not amplify with An. arabiensis, An. coluzzi, or An. gambaie s.s. primers are listed as *An. gambiae s.l.*
3. Other species identified include *An. stephensi* (n=3), *An. rufipes* (n=2), *An. coustani* (n=4), and *An. demeilloni* (n=7). None had *P.f*. or human bloodmeal detected. Two *An. coustani* and one *An. stephensi* were positive for non-human bloodmeal

## Discussion

Malaria ecology is unique in northern Kenya. Transmission is shaped by the arid environment, sparse rainfall, and seasonal rivers. Prior to 2024, mosquito populations were naive to vector control. Thus, we were able to document species composition, bloodmeal sources and *P.falciparum* infection before significant vector control activities. *Anopheles* population density was highly seasonal with a major peak in September that corresponded to an atypically high transmission season (Asibitar, 2024). During some months, no mosquitoes - anopheline or non-anopheline - were observed at all. Overall, we collected 10 times more mosquitoes in the rural area with the same trapping effort, even though both areas have substantial malaria case burden as reported by the local health facilities.

We observed a diverse mix of potential vector species, notably a substantial proportion of *An. pharoensis* and *An. coluzzii*. *An. coluzzii*, a sibling species in the *An. gambaie* complex, is widely distributed in West Africa (De Marco et al., 2025), but has only recently been identified in one other report in East Africa, also from Turkana (Kamau et al., 2024), despite the fact that ecological modeling studies predicts that Kenya is poorly suited for *An. coluzzii* (De Marco et al., 2025). Most reports from West Africa describe *An. gambaie s.s.* and *An. coluzzii* as geographically sympatric (Ahoua Alou et al., 2025; Ajayi et al., 2025; Niang et al., 2014; Opondo et al., 2025; Yovogan et al., 2025) although with differing preferences for breeding habitats. However, we did not find any *An. gambaie s.s.* in our study area, possibly due to low humidity whereas its sibling species, *An. arabiensis*, is more tolerant to drier climate (Koenraadt et al., 2004). *An. gambiae s.s*. is typically highly anthropophilic relative to the species we observed in Turkana, possibly reflecting how vector diversity is shaped by the close proximity of humans and livestock, with livestock significantly outnumbering humans. Although the invasive vector species, *An. stephensi*, has been identified in Turkana (Ochomo et al., 2023) and is abundant in neighboring Ethiopia (Balkew et al., 2021), we observed very few *An. stephensi* indicating that intense local transmission is not dependent on the emergence of this species.

All the vector species we identified were confirmed to feed on humans and transmit malaria as indicated by the presence of *P. falciparum*. *An. coluzzii* harboured double the prevalence of parasites compared to *An. arabiensis*. Studies in Niger (Moustapha et al., 2025) and Cote d’Ivoire (Gouamene et al., 2024) also demonstrated high *P. falciparum* infection in *An coluzzii* compared to other vector species. Interestingly, although more mosquitoes were collected indoors, the parasite rate was higher in outdoor collected mosquitoes. This could be attributed to the fact that people in Turkana prefer to sleep outdoors. A quarter of female anopheles had non-human bloodmeals and the proportion was similar across indoor and outdoor collections, possibly due to the continuous nature of indoor versus outdoor particularly in rural areas where homes are completely permeable to mosquitoes.

This study has several limitations to be weighed. First, we implemented passive, baited trapping which results in catches enriched in unfed, host-seeking insects. This limits our ability to describe the preferred hosts and feeding success of these vectors. Second, the bloodmeal analysis only differentiated human and non-human sources, preventing us from identifying which hosts are sustaining vector populations. Despite these limitations, our findings have important implications for vector control efforts in the region. First, unlike other areas in the southern part of Kenya (Bayoh et al., 2010), the pre-ITN vector population was predominantly *An. arabiensis* with no *An. gambiae s.s*. observed across the study period. This prevents us from extrapolating the impact of ITNs observed in other areas to Turkana. Second, the overall vector population density may not be affected by ITNs since the species identified do not depend heavily on human sources of blood. Although ITNs may afford individual protection to the user, population-level protection due to the lethal effect on host-seeking mosquitoes may not be significant in this context. It should also be noted that these vector species have been reported to harbor significant resistance to pyrethroids in Kenya and elsewhere (Correa et al., 2024; Hancock et al., 2018; Opondo et al., 2025; Wiebe et al., 2017; Yovogan et al., 2025) which could further diminish the effect of ITNs. Finally, mosquitoes collected outdoors were more likely to be infected suggesting that a significant amount of transmission is occurring outdoors. This is not surprising since most households have at least one outdoor sleeping space. Vector control approaches such as IRS and ITNs may not be optimal for this context. Expectations for the impact of ITNs on malaria in the region should be tempered by these findings.

## Supplementary Material

Supplementary Files

This is a list of supplementary files associated with this preprint. Click to download.

• SupplementaryTables.docx

## Figures and Tables

**Table 1:** *P. falciparum* infection and bloodmeal by trap location

	Trap location			
	Indoors	Outdoors	Total	p-value
N	2,012 (80.4%)	490 (19.6%)	2,502 (100.0%)	
***P. falciparum***				
uninfected	1,872 (96.0%)	451 (93.8%)	2,323 (95.5%)	0.056
infected	83 (4.1%)	30 (6.1%)	113 (4.5%)	
**Bloodmeal**				
Human bloodmeal	39 (21.9%)	14 (2.9%)	53 (2.1%)	0.073
Non-human bloodmeal	427 (21.2%)	123 (25.1%)	550 (22.0%)	
Unfed	1,485 (73.8%)	344 (70.2%)	1,829 (73.1%)	
Undetermined	61 (3.0%)	9 (1.8%)	70 (2.8%)	

## Data Availability

The datasets used and/or analyzed during the current study are available from the corresponding author on reasonable request.
